# Biomarkers and cytokines of bone turnover: extensive evaluation in a cohort of patients with ankylosing spondylitis

**DOI:** 10.1186/1471-2474-13-191

**Published:** 2012-10-02

**Authors:** Ali Taylan, Ismail Sari, Baris Akinci, Safak Bilge, Didem Kozaci, Servet Akar, Ayfer Colak, Hulya Yalcin, Necati Gunay, Nurullah Akkoc

**Affiliations:** 1 Izmir Tepecik Training and Research Hospital, Department of Rheumatology, Izmir, Turkey; 2 Dokuz Eylul University School of Medicine, Department of Internal Medicine, Division of Rheumatology, Izmir, Turkey; 3 Izmir Tepecik Training and Research Hospital, Department of Endocrinology, Izmir, Turkey; 4 Izmir Tepecik Training and Research Hospital, Department of Physical medicine and Rehabilitation, Izmir, Turkey; 5 Adnan Menderes University School of Medicine, Department of Biochemistry, Aydin, Turkey; 6 Izmir Tepecik Training and Research Hospital, Department of Biochemistry, Izmir, Turkey; 7 Adnan Menderes University, Bilim ve Teknoloji Araştirma ve Uygulama Merkezi (ADU-BILTEM), Aydin, Turkey

**Keywords:** Ankylosing spondylitis, Bone mineral density, OPG, RANKL, DKK-1

## Abstract

**Background:**

Ankylosing spondylitis (AS) is a chronic inflammatory disease of spine and sacroiliac joints; it is characterized by new bone formation, and the disease processes can be accompanied by osteoporosis. In the present study, we investigated changes in bone mineral density (BMD) and in the levels of various bone turnover-related biomarkers and cytokines in a cohort of AS patients, with regard to clinical parameters, disease activity, and treatment regimen.

**Methods:**

55 AS patients and 33 healthy controls included in the study. Spinal mobility was assessed by the Bath Ankylosing Spondylitis Metrology Index (BASMI), and radiologic changes were scored by the Bath Ankylosing Spondylitis Radiologic Index (BASRI). Patients were also evaluated with the Bath Ankylosing Spondylitis Functional Index (BASFI) and the Bath Ankylosing Spondylitis Disease Activity Index (BASDAI). Bone mineral density (BMD) assessed by dual energy X-ray absorptiometry. Various biomarkers and cytokines of bone turnover including osteoprotegerin (OPG), serum band 5 tartrate-resistant acid phosphatase (TRAP-5), soluble receptor activator of nuclear factor kappa-B ligand (sRANKL), secreted frizzled-related protein 1 (sFRP-1), Dickkopf-related protein 1 (DKK-1), and sclerostin were studied.

**Results:**

The levels of TRAP-5, NTX, sRANKL, sclerostin, sFRP-1, DKK-1, and IFNγ, were similar between the patients and controls (*p* > 0.05), while BMD of femoral neck, and OPG levels were significantly lower in AS patients (*p* < 0.05). In a subgroup analysis, patients with active disease had significantly higher concentrations of OPG compared with the inactive group. Rest of the biomarkers and cytokines of bone turnover were similar between the active and inactive disease groups. Subgroup analysis of patients receiving anti-TNFα agents and conventional therapy revealed that OPG concentrations were significantly lower in the patients receiving biological drugs, while BAP and DKK-1 were significantly higher in the patients treated with conventional agents.

**Conclusions:**

In this cross-sectional study we showed that OPG levels were significantly lower in AS patients compared to healthy subjects. On the other hand, the levels of wingless (Wnt) signal pathway inhibitors seem not altered. Ectopic bone formation in AS may be related to dysfunction of these molecules at the cellular level.

## Background

Ankylosing spondylitis (AS), a chronic inflammatory disease of the spine and sacroiliac joints, is characterized by new bone formation and is thereby associated with syndesmophytes and ankylosis [1]. Osteoporosis is also present in AS and has multiple etiologies [2,3]. Several biomarkers are known to be related to bone turnover. In general, activation of osteoclasts (OCs) plays an important role in bone loss and the development of erosions. Human receptor activator of nuclear factor-κβ ligand (RANKL), a member of the tumor necrosis factor super family, is one molecule that stimulates OCs. In contrast, inhibition of OCs and activation of osteoblasts (OBs) are linked with new bone formation and ossification. Osteoprotegerin (OPG), which is produced by OBs, inhibits RANKL and plays an important role in bone formation [4]. Recently, various components of the Wnt pathway signaling cascade were found to be involved in maintaining bone mass. The most-studied secreted Wnt inhibitors are sclerostin, dickkopfs (Dkks), and secreted frizzled related proteins (sFRP1), which likely play important roles in bone turnover [5].

Various studies have aimed to identify and clarify the mechanisms of bone turnover in AS, which results in new bone formation and, paradoxically, osteoporosis [6-13]. The use of anti-TNF treatments has provided new insights into the treatment of inflammatory rheumatic diseases, particularly AS and rheumatoid arthritis (RA). Current data suggested that formation of syndesmophytes is not prevented by anti-TNF treatments, despite significant clinical improvement [14]. It is not known why bone formation continues even when inflammation is suppressed [14,15]; therefore; further studies are needed to identify pathophysiological mechanisms responsible for new bone formation and osteoporosis in AS. In the current study, we evaluated the biomarkers and cytokines related to bone turnover in AS patients who were treated with conventional agents and anti-TNFs. We also investigated the relationship of bone mineral density with various biomarkers of bone turnover and clinical parameters.

## Methods

Fifty-five consecutive patients diagnosed with AS according to modified New York criteria [16], and 33 age- and sex-matched healthy controls were enrolled in this study. Subjects with a history of neuroendocrine disorder (thyroid, parathyroid disorder, and anticonvulsant usage), chronic renal and liver disease, systemic steroid usage, menopause, cigarette smoking, or excessive alcohol usage were excluded from the study.

Patients provided informed written consent to participate, and the study protocol was approved by the ethical committee of the Tepecik Hospital. Spinal mobility was assessed by the Bath Ankylosing Spondylitis Metrology Index (BASMI) [17]. Radiologic changes were scored by the Bath Ankylosing Spondylitis Radiologic Index (BASRI) [18]. Patients were also evaluated with the Bath Ankylosing Spondylitis Functional Index (BASFI) [19] and the Bath Ankylosing Spondylitis Disease Activity Index (BASDAI) [20]. Patients with a BASDAI ≥ 4 were defined as having active disease. Conventional treatment included non-steroidal anti-inflammatory drugs (NSAIDs) and/or sulfasalazine, but not anti-TNF-α drugs.

### Laboratory evaluation

Following an overnight fast, venous blood samples for laboratory tests were collected between 8:00 and 9:00 AM. Serum samples were preserved at *−*80°C until assayed. The following ELISA kits were used according to the manufacturer’s instructions: Serum high sensitive C- reactive protein (hs-CRP, Cat. No.: EIA-3954, DRG International, USA), interleukin 6 (IL-6, Cat. No.: EK0410, Boster Biological Technology Co., Ltd., China), vitamin D (Cat. No.: K 2110 ko, ImmunDiagnostik, Bensheim, Germany), bone-specific alkaline phosphatase (BAP, QUIDEL, San Diego, CA, USA), cross-linked N-telopeptide of type I collagen (NTx, Osteomark® NTx serum, Princeton, NJ, USA), serum band 5 tartrate-resistant acid phosphatase (TRAP-5, Cat. No.: RD197025000, Biovendor Laboratory Medicine, Brno, Czech Republic), soluble receptor activator of nuclear factor kappa-B ligand (sRANKL, Cat. No.: RD193004200R, Biovendor Laboratory Medicine, Brno, Czech Republic), osteoprotegerin (OPG, Cat. No.: EK0480, Boster Biological Technology Co., Ltd., China), sclerostin (Cat. No.: BI-20492, Biomedica Gruppe, Vienna, Austria), Dickkopf-related protein 1 (DKK-1, Cat. No.: BI-20412, Biomedica Gruppe, Vienna, Austria), secreted frizzled-related protein 1 (sFRP-1, Cat. No.: E95880Hu, USCN, Wuhan, China), human parathyroid hormone (hPTH, Cat. No.: KAP1481, The DIAsource hPTH-EASIA, Niveles, Belgium) serum interferon-gamma (IFN-γ, Cat. No.: EI1023-1, AssayPro, St. Charles, MO, USA).

### Bone densitometry assessment

Axial and appendicular BMD of lumbar spine (L2-L4, posteroanterior view) and the left proximal femur (neck, intertrochanteric area, greater trochanter, and ward’s triangle) were assessed using the AP dual-energy x-ray absorptiometry DEXA (Hologic QDR 1000, Waltham, MA, USA). The results were expressed as g/cm^2^.

### Statistical analysis

The Kolmogorov-Smirnov normality test was used to determine the distribution pattern of the variables. The majority of the parameters including ESR, BAP, DKK-1, hPTH, IL-6, sFRP1, IFN-γ, and sRANKL/OPG ratio showed non-normal distribution, and we used non-parametric tests for the statistical analysis. The Mann–Whitney *U*-test was used for comparisons between two groups of continuous variables. Fisher's exact test was performed for the comparison of categorical variables. The Spearman’s rho correlation was used to determine relationships between parameters. Results were presented as median with minimum–maximum (range) values. Statistical analysis was carried out using the Statistical Package for the Social Science, version 13.0 (Chicago, IL, USA). A two-tailed *p* value of <0.05 was considered significant.

## Results

Of the 55 AS patients, 48 were male, 7 were female, and the median age was 36 years (range, 19–61); of the 33 healthy controls, 24 were male, 9 were female, and the median age was 39 years (range, 23–48). In the patient group, the median disease duration was 10 (2–40) years. BASDAI, BASFI, BASMI, BASRI hip, and BASRI spine indices were 5 (1–9), 3.7 (0.1–8.8), 3 (1–9), 0 (0–4), and 6 (2–12), respectively. HLAB-27 positivity was 64.9%. None of the individuals had any personal or family history of psoriasis or inflammatory bowel disease. Only one patient had a history of hip prosthesis.

Age and sex distributions were similar between patients and controls (*p* = non-significant [NS]). Anthropometrical parameters, including body mass index (BMI) and waist circumference, were also comparable between the groups (*p* = NS). Biochemical examination showed that serum levels of calcium, phosphor, BAP, and vitamin D did not differ between patients and controls (*p* = NS). However, acute phase proteins, including ESR, hs-CRP, and IL-6, were significantly higher in the AS group (*p* < 0.05). Bone turnover parameters, including TRAP 5, NTX, sRANKL, sclerostin, sFRP-1, DKK-1, and IFNγ, were similar between the groups (*p* = NS). On the other hand, hPTH and OPG levels were significantly lower in the AS group [*p* < 0.05; 46 (44–170) vs. 78 (45–180) pg/mL and 339 (52–1118) vs. 527 (16–1030) pg/mL, respectively]. BMD of femoral neck in the patient group was significantly lower than in the controls [*p* < 0.05; 0.8 (0.5–1) vs. 0.9 (0.6–1.1) g/cm^2^]. However, BMD lumbar spine did not differ between the groups (*p* = NS). The clinical and laboratory characteristics of AS patients and controls are given in Table 1.

**Table 1:** Clinical and laboratory characteristics of AS patients and controls

	**AS Patients (n =55)**	**Controls (n = 33)**	***p*value**
**Age (years)**	36 (19–61)	39 (23–48)	0.9
**Sex (M/F)**	48/7	24/9	0.1
**BMI (kg/m**^**2**^**)**	25 (18–37)	25 (20–31)	0.9
**Waist circumference (cm)**	92 (66–120)	89 (65–104)	0.2
**BMD-Lumbar spine (g/cm2)**	0.9 (0.7–1.6)	1 (0.8–1.2)	0.1
**BMD-Femoral neck (g/cm2)**	0.8 (0.5–1.1)	0.9 (0.6–1.1)	0.002
**ESR (mm/h)**	17 (3–72)	9 (1–30)	<0.0001
**hs-CRP (mg/L)**	8.5 (0.6–24.3)	2.5 (0–9.9)	<0.0001
**IL-6 (pg/mL)**	18.5 (5–545)	8.1 (4–69)	<0.0001
**IFNγ (pg/mL)**	0.002 (0.002–1.956)	0.002 (0.002–0.313)	0.5
**BAP (U/L)**	29 (13–107)	26 (2–51)	0.1
**Calcium (mg/L)**	10 (9–11)	9.9 (9.3–11)	0.7
**Phosphor (mg/dL)**	1.3 (1.7–4.7)	3.4 (2.2–4,5)	0.9
**Vitamin D (nM)**	87 (4–266)	102 (10–303)	0.5
**NTX (nM, bone collagenequivalent)**	82 (52–188)	84 (57–106)	0.3
**TRAP 5 (U/L)**	3.8 (2.3–6.6)	3.7 (2.3–8.34)	0.3
**hPTH (pg/mL)**	46 (44–170)	78 (45–180)	0.02
**sRANKL (pmol/L)**	284 (101–1598)	292 (143–783)	0.8
**OPG (pg/mL)**	339 (52–1118)	527 (16–1030)	0.02
**sRANKL/OPG**	0.99 (0.14-22.6)	0.65 (0.14–25.1)	0.04
**Sclerostin (pmol/L)**	73 (48–126)	77 (49–110)	0.2
**sFRP1 (ng/mL)**	0.14 (0.1–16.7)	0.2 (0.1–1.2)	0.4
**DKK1 (pg/mL)**	97 (17–771)	115 (29–278)	0.7

Data are presented as the median with minimum and maximum values. ESR= erythrocyte sedimentation rate, hs-CRP= high sensitive C- reactive protein, IL-6= interleukin 6, IFNγ= interferon-gamma, BAP= bone-specific alkaline phosphatase, NTX= cross-linked N-telopeptide of type I collagen, TRAP 5= serum band 5 tartrate-resistant acid phosphatase, hPTH= human parathyroid hormone, sRANKL= soluble receptor activator of nuclear factor kappa-B ligand, OPG= osteoprotegerin, sFRP1= secreted frizzled-related protein 1, DKK1= Dickkopf-related protein 1.

### Comparison of AS subjects receiving anti-TNF agents and conventional therapy

Thirty-two patients were treated with conventional drugs (NSAIDS and/or sulfasalazine), and 23 were treated with anti-TNFα drugs (9 infliximab, 8 etanercept, and 6 adalimumab). Age distribution was similar between the groups (*p* = NS). The median treatment duration of biologics was 24 (16–33) months. All subjects treated with TNFα-targeting agents received their treatments regularly, and none of the patients treated with conventional drugs had received anti-TNFα agents in their past medical history. Comparison of the patients on anti-TNFα and conventional drug therapies revealed that all biochemical parameters were similar in both treatment groups (*p* = NS), except for BAP, DKK-1, and OPG. BAP and DKK-1 concentrations were significantly higher in the patients receiving anti-TNFα drugs [*p* < 0.05; 33 (17–107) vs. 28 (13–47) U/L, and 108 (39–771) vs. 87 (17–370), pg/mL respectively], while OPG levels were lower [*p* < 0.05; 274 (52–1080) vs. 384 (84–1118) pg/mL respectively] in the anti-TNFα treatment group compared to conventional therapy. BASRI-hip and -spine indices and BMD assessments (lumbar and femoral neck) did not differ between the treatment groups (*p* = NS). Table 2 summarizes the parameters of the two treatment groups.

**Table 2:** **Comparison of AS subjectswith respect to treatmenttype**

	**Anti-TNF treatment(n = 23)**	**Conventional treatment(n = 32)**	***p*value**
**BASFI**	3.7 (1.5–7.7)	3.8 (0.1–8.8)	0.4
**BASDAI**	4 (1.3–8.1)	5.5 (1–9)	0.08
**BASRI-hip**	0 (0–4)	0 (0–2.5)	0.1
**BASRI-spine**	7.5 (2–12)	5.5 (2–12)	0.09
**BASMI**	4 (1–9)	2 (1–9)	0.003
**BMD-Lumbar spine (g/cm2)**	1 (0.7–1.6)	0.9 (0.8–1.4)	0.3
**BMD-Femoral neck (g/cm2)**	0.8 (0.6–1)	0.8 (0.5–1.1)	0.5
**IL-6 (pg/mL)**	13 (5–545)	20 (6–300)	0.9
**IFNγ (pg/ml)**	0.002 (0.002–0.03)	0.002 (0.002–1.96)	0.6
**BAP (U/L)**	33 (17–107)	28 (13–47)	0.04
**Vitamin D (nM)**	84 (12–266)	92 (4–244)	0.9
**NTX (nM, bone collagenequivalent)**	78 (52–188)	83 (64–114)	0.4
**TRAP 5 (U/L)**	4 (2.3–5.8)	3.7 (2.3–6.6)	0.3
**hPTH (pg/mL)**	46 (44–130)	46 (44–170)	0.8
**sRANKL (pmol/L)**	284 (103–1598)	285 (101–956)	0.5
**OPG (pg/mL)**	274 (52–1080)	384 (84–1118)	0.04
**sRANKL/OPG**	1.28 (0.2–22.6)	0.73 (0.14–4.5)	0.03
**Sclerostin (pmol/L)**	75 (48–95)	68 (49–126)	0.6
**sFRP1 (ng/mL)**	0.1 (0.1–17)	0.2 (0.1–0.4)	0.1
**DKK1 (pg/mL)**	108 (39–771)	87 (17–370)	0.03

Data are presented as the median with minimum and maximum values. BASFI= Bath ankylosing apondylitis functional index, BASDAI= Bath ankylosing spondylitis disease activity index, BASMI= Bath ankylosing spondylitis metrology index, BASRI= Bath Ankylosing spondylitis radiologic index, IL-6= interleukin 6, IFNγ= interferon-gamma, BAP= bone-specific alkaline phosphatase, NTX= cross-linked N-telopeptide of type I collagen, TRAP 5= serum band 5 tartrate-resistant acid phosphatase, hPTH= human parathyroid hormone, sRANKL= soluble receptor activator of nuclear factor kappa-B ligand, OPG= osteoprotegerin, sFRP1= secreted frizzled-related protein 1, DKK1= Dickkopf-related protein 1.

### Comparison of AS subjects with respect to disease activity

Thirty-eight AS patients had active disease (BASDAI ≥ 4), and 17 cases were inactive. Age and sex distributions were similar between patients with active and inactive disease (*p* = NS). Studied bone turnover markers, except OPG, were comparable between active and inactive disease states (*p* = NS). OPG concentrations were significantly higher in the active AS group compared to the inactive patients [*p* < 0.05; 367 (52–1118) vs. 274 (71–592) pg/mL). BASRI-hip and -spine indices and BMD assessments (lumbar and femoral neck) were also not different between active and inactive patients (*p* = NS). Data from patients with active and inactive diseases are shown in Table 3.

**Table 3:** Comparison of AS subjects with respect to disease activity

	**Active patients(BASDAI ≥ 4; n= 38)**	**Inactive patients(BASDAI < 4; n= 17)**	***p*value**
**IL-6 (pg/mL)**	20 (6–545)	15 (5–277)	0.2
**IFNγ (pg/mL)**	0.002 (0.002–1.96)	0.002 (0.002–0.89)	0.7
**BAP (U/L)**	28 (13–107)	30 (15–49)	0.3
**Vitamin D (nM)**	89 (4–266)	77 (12–240)	0.6
**NTX (nM, bone collagenequivalent)**	87 (56–131)	81 (52–188)	0.7
**TRAP 5 (U/L)**	3.7 (2.2–5.8)	3.9 (2.4–6.6)	0.4
**hPTH (pg/mL)**	46 (44–170)	77 (45–130)	0.08
**sRANKL (pmol/L)**	264 (101–956)	350 (158–1598)	0.2
**OPG (pg/mL)**	367 (52–1118)	274 (71–592)	0.004
**sRANKL/OPG**	0.79 (0.14–4.5)	1.16 (0.4–22.6)	0.02
**Sclerostin (pmol/L)**	70 (49–126)	75 (48–123)	0.7
**sFRP1 (ng/mL)**	0.15 (0.1–17)	0.1 (0.1–0.2)	0.5
**DKK1 (pg/mL)**	93 (26–370)	130 (17–771)	0.5
**BASRI-hip**	0 (0–4)	0.5 (0–4)	0.2
**BASRI-spine**	5.5 (2–12)	7 (2–12)	0.3
**BASMI**	4 (1–9)	2.5 (1–9)	0.7
**BMD-Lumbar spine (g/cm2)**	0.9 (0.7–1.4)	0.9 (0.7–1.6)	0.8
**BMD-Femoral neck (g/cm2)**	0.7 (0.6–1.1)	0.8 (0.5–1)	0.9

Data are presented as the median with minimum and maximum values. BASDAI= Bath ankylosing spondylitis disease activity index, IL-6= interleukin 6, IFNγ= interferon-gamma, BAP= bone-specific alkaline phosphatase, NTX= cross-linked N-telopeptide of type I collagen, TRAP 5= serum band 5 tartrate-resistant acid phosphatase, hPTH= human parathyroid hormone, sRANKL= soluble receptor activator of nuclear factor kappa-B ligand, OPG= osteoprotegerin, sFRP1= secreted frizzled-related protein 1, DKK1= Dickkopf-related protein 1, BASRI= Bath Ankylosing spondylitis radiologic index, BASMI= Bath ankylosing spondylitis metrology index.

### Correlation analysis

Upon correlation analysis, sclerostin was found to be correlated with disease duration, lumbar BMD, DKK-1, and PTH (p < 0.05; r = 0.3, 0.2, 0.2, and 0.3, respectively). DKK-1 showed correlations with BASMI, sclerostin, and SFRP-1 (p < 0.05; r = 0.3, 0.2, and −0.2, respectively). sRANKL was correlated with disease duration, BASRI-spine, and BASMI (p < 0.05; r = −0.3, −0.4, and −0.4 respectively). Lumbar BMD was correlated with disease duration, BASRI-spine, femoral neck BMD, BASMI, and sclerostin (p < 0.05; r = 0.3, 0.3, 0.3, 0.4, and 0.2 respectively). OPG showed correlations with BASDAI ≥ 4 (p < 0.05; r = 0.3). Other significant correlations were summarized in Table 4.

**Table 4:** Correlation coefficients of the some clinical and laboratory data

	**Disease duration**	**BASRI-h**	**BASRI-s**	**BMD-f**	**BMD-l**	**BASDAI ≥4**	**BASMI**	**hsCRP**	**OPG**	**sRANKL**	**Sclerostin**	**DKK1**	**sFRP1**
Disease duration		0.3	0.6		0.3		0.6			−0.3	0.3		
BASRI-h	0.3		0.6			−0.3	0.5	0.4					
BASRI-s	0.6	0.6			0.3		0.7	0.3		−0.4			
BMD-f					0.3								
BMD-l	0.3		0.3	0.3			0.4				0.2		
BASDAI ≥ 4		−0.3							0.3				
BASMI	0.6	0.5	0.7		0.4					−0.4		0.3	
hsCRP		0.4	0.3										
OPG						0.3							
sRANKL	−0.3		−0.4				−0.4						−0.2
Sclerostin	0.3				0.2							0.2	
DKK1							0.3				0.2		−0.2
sFRP1												−0.2	

Values indicate correlation coefficients. Note that empthy cells indicate no significance between variables. BASRI-h, and BASRI-s= Bath ankylosing spondylitis radiology index hip, and spine respectively, BMD-f, and BMD-l= BMD femoral neck and lumbar spine respectively, BASFI= Bath ankylosing apondylitis functional index, BASDAI= Bath ankylosing spondylitis disease activity index, BASMI= Bath ankylosing spondylitis metrology index, sRANKL= soluble receptor activator of nuclear factor kappa-B ligand, OPG= osteoprotegerin, sFRP1= secreted frizzled-related protein 1, DKK1= Dickkopf-related protein 1.

## Discussion

The present study showed that AS patients had lower serum OPG levels and a higher RANKL/OPG ratio compared to healthy controls. Previous studies have shown discrepancies in regards to OPG level in AS. Some studies revealed elevated OPG levels in AS patients compared to healthy controls [6,9,12]. However, a study in a Korean cohort found no differences in serum levels of OPG between AS patients and normal subjects [11]. In a cohort of 240 AS patients, Franck *et al.* found that AS patients generally had lower serum OPG level and higher RANKL/OPG ratio; they also reported a lack of compensatory increase of OPG with age in AS patients [8]. Subgroup analysis of our results also revealed that active disease state (BASDAI ≥ 4) was associated with higher OPG levels. This result is in agreement with the findings of Chen *et al*., who found OPG to be correlated with poor physical mobility and increased inflammatory state in AS patients; however, in contrast to our results, that study found RANKL and OPG to be elevated compared to controls [6]. Osteoprotegerin inhibits osteoclastogenesis by binding RANKL, acting as a decoy receptor to competitively inhibit RANKL interaction with its receptor RANK. Thus, decreased OPG levels in AS patients might have contributed to the lowered BMD detected at femur in our study group [4]. On the other hand, hypothetically, the excess production of OPG during active disease state could contribute to the new bone formation. In this study, we also showed that, anti-TNF treated AS patients had significantly lower levels of OPG compared with the conventionally treated patients. This observation is in line with a prospective study performed by Kwon et al. who showed anti-TNF treatment had a negative effect on OPG concentrations [12].

The discrepancies between the results of various studies may be due to the effects of various disease states and treatment types on the RANKL/OPG axis, as well as the complex interaction of various cytokines and signaling molecules. In our study, RANKL was similar in AS patients and healthy controls, was not altered with treatment, and showed no relation with clinical parameters; in contrast, Kim *et al*. found increased RANKL level correlating with disease activity markers [11]. RANKL is an important cytokine, but osteoclastogenesis is also influenced by TNF, IL-1, IL-6, IL-17, vitamin D3, and prostaglandin E2 [21,22]. OPG balances bone resorption, and is induced by IL-4, IFNγ, PTH, BMP, and Wnt signaling [23]; all of these cytokines are expressed differently in cases of certain disease activity and treatment. Woo *et al*. reported that etanercept therapy didn’t change RANKL/OPG levels, but improved bone metabolic markers and BMD in AS patients [24]. In our study, we observed that sRANKL/OPG ratio was significantly higher in patients treated with anti-TNF therapies. Although sRANKL did not change between the treatments groups, this significance was a consequence of lower OPG concentrations in the biological treatment group. The effects of inflammation on bone remodeling have different outcomes in different rheumatic diseases, such as RA and AS [25]. In both diseases, decreased bone mineral density is expected, including juxta-articular osteopenia in RA and decreased BMD mainly at vertebras and hip in AS [2,3]. In this study, BMD of femoral neck was significantly decreased in AS patients, but BMD of lumbar spine did not differ from the healthy controls. These observations may be due to be the interference of syndesmophytes and ectopic calcifications with BMD measurement at lumbar spine. Low PTH and OPG in AS patients may be explanatory to the finding of decreased BMD of femoral neck as we noted previously.

In our study, the serum levels of Wnt signaling pathway inhibitors, DKK-1, Sclerostin, and sFRP1, were not different in AS patients from the healthy controls. However, subgroup analysis showed that patients receiving anti-TNFα treatment had higher DKK-1 levels compared to patients taking conventional drugs. Daoussis *et al*. recently measured DKK-1 level using two different serum assays; a sandwich Elisa assay that showed circulating DKK-1 indicated that serum DKK-1 was increased in AS patients compared to healthy controls, but a functional ELISA model showed decreased binding of DKK-1 to the LRP-6 receptor [7]. Diarra *et al.* also found that, in contrast to RA, DKK-1 levels in AS were very low and showed no relation with measures of disease activity [26]. Lane *et al*. found that higher DKK-1 levels were protective and diminished the risk of radiologic progression for hip OA, another disease associated with bone formation [27]. All of these studies suggest that either dysfunction or decreased level of DKK-1 is crucial for new bone formation in AS. In our study, increased DKK-1 level in AS patients who were on anti-TNF treatment are noteworthy, since the balance between DKK-1 and Wnt is important for bone turnover. In Diarra *et al*.’s study, DKK-1 levels in RA patients were diminished with anti-TNF drugs, but the effects in AS patients were not clear [26]. According to our results, decreased OPG and increased DKK-1 in patients on anti-TNF therapy suggest a trend favoring osteoclastogenesis in these patients. However, prospective studies are needed to determine the net effect of biological therapies on bone metabolism.

## Conclusions

In this cross-sectional study we showed that OPG levels were significantly down regulated in AS patients compared to healthy subjects. Serum concentrations of OPG tend to be higher in patients with active disease state suggesting a trend favoring osteoblastic activity in these patients. The levels of Wnt signal pathway inhibitors seem not altered in AS. Ongoing ectopic bone formation, one of the one of the hallmark features of AS, may be related to dysfunction of these molecules at the cellular level.

## Competing interests

The authors declare that they have no competing interests.

## Authors’ contributions

DK, AC, HY and NG are carried out all the laboratory analysis in the study. SA read x-rays for BASRI calculation. SB helped to collect data from patients. IS did statical analysis of the results and contributed to discussion, also involved intellectually in project design. NA helped to general design of the paper and supported us, as a head of rheumatology department. BA involved in project design intellectually. AT involved in project design and collected the patient’s data and wrote the paper. All authors read and approved the final manuscript.

## Pre-publication history

The pre-publication history for this paper can be accessed here:

http://www.biomedcentral.com/1471-2474/13/191/prepub
